# Reprogramming of cassava (*Manihot esculenta*) microspores towards sporophytic development

**DOI:** 10.1093/aobpla/plu022

**Published:** 2014-05-21

**Authors:** P. I. P. Perera, C. A. Ordoñez, B. Dedicova, P. E. M. Ortega

**Affiliations:** 1 Agrobiodiversity Research Area, International Center for Tropical Agriculture, A.A. 6713, Cali, Colombia; 2 Present address: Department of Horticulture and Landscape Gardening, Faculty of Agriculture and Plantation Management, Wayamba University, Gonawila, Makandura, Sri Lanka; 3 National University of Colombia, Palmira, Colombia

**Keywords:** Androgenesis, DAPI, histology, *Manihot esculenta*, reprogramming, scanning electron microscopy, transmission electron microscopy.

## Abstract

Gametes have the unique potential to enter the sporophytic pathway, called androgenesis. The plants produced are usually haploid and recombinant due to the preceding meiosis and they can double their chromosome number to form doubled haploids, which are completely homozygous. Availability of the doubled haploids facilitates mapping the genes of agronomically important traits, shortening the time of the breeding process required to produce new hybrids and homozygous varieties, and saving the time and cost for inbreeding. This study aimed to test the feasibility of using isolated and *in vitro* cultured immature cassava (*Manihot esculenta*) microspores to reprogramme and initiate sporophytic development. Different culture media and different concentrations of two ion components (Cu^2+^ and Fe^2+^) were tested in two genotypes of cassava. External structural changes, nuclear divisions and cellular changes during reprogramming were analysed by scanning electron microscopy, by staining with 4′,6-diamidino-2-phenylindole, and through classical histology and transmission electron microscopy. In two cassava genotypes, different developmental stages of microspores were found to initiate sporophytic cell divisions, that is, with tetrads of TMS 60444 and with mid or late uni-nucleate microspores of SM 1219-9. In the modified NLN medium (NLNS), microspore enlargements were observed. The medium supplemented with either sodium ferrous ethylene-diamine-tetraacetic acid (NaFeEDTA) or CuSO_4_·5H_2_O induced sporophytic cell division in both genotypes. A low frequency of the reprogramming and the presence of non-responsive microspores among the responsive ones in tetrads were found to be related to the viability and exine formation of the microspores. The present study clearly demonstrated that reprogramming occurs much faster in isolated microspore culture than in anther culture. This paves the way for the development of an efficient technique for the production of homozygous lines in cassava. This is the first ever detailed report of microspore reprogramming at the tetrad stage and the first report of microspore embryogenesis induction in cassava with detailed evidence.

## Abstract (precis)

Gametes have a unique potential to enter the sporophytic pathway, called androgenesis. The plants produced are usually haploid and recombinant due to the preceding meiosis and they can double their chromosome number to form doubled haploids (DHs), which are completely homozygous. The present study demonstrates that reprogramming occurs in the isolated microspores at tetrad stage as the first step if androgenesis and this is paving the way for the development of an efficient technique for the production of homozygous lines in cassava. This is the first ever detailed report of microspore reprogramming at the tetrad stage and the first report of microspore embryogenesis induction in cassava with detailed evidence.

## Introduction

The life cycle of plants consists of a very long sporophytic phase and a much shorter gametophytic phase. Cells of the latter phase have the unique potential to enter the sporophytic pathway, i.e. embryogenesis, without fertilization once they are reprogrammed by appropriate triggers and cultured *in vitro* (Forster *et al.* 2007). This fascinating example of phase transition during the alternation of generations in plants is called androgenesis (Raghavan 1986). The plants produced are usually haploid and recombinant due to the preceding meiosis, but they can double their chromosome number to form doubled haploids, which are completely homozygous. Efficient methods to establish doubled haploid lines are essential for researchers to map genes of agronomically important traits as well as for breeders to shorten the time of the breeding process required to produce new hybrids and homozygous varieties. The introduction of an efficient doubled haploid production system saves time and cost over conventional inbreeding.

Although anther culture is the most common technique for the production of haploid plants, microspore culture is more efficient since thousands of microspores can be cultured on a single tissue culture plate. Moreover, microspore culture avoids the formation of calli or embryos from somatic tissues of the anthers as reported in cassava (*Manihot esculenta*) (Perera *et al.* 2014), giving rise only to plants that are haploids or doubled haploids. Direct access to the microspores speeds up the optimization of culture conditions, and the entire process of microspore embryogenesis can easily be monitored under an inverted microscope (Polsoni *et al.* 1988; Graner 1996). High frequencies of embryo formation and plantlet regeneration in isolated microspore cultures have been obtained in only a few plant species, such as tobacco, rapeseed, barley, wheat and rice (Jähne and Lörz 1995; Palmer *et al.* 1996; Touraev and Heberle-Bors 2003), even though microspore embryogenesis has been reported to occur in more than 250 plant species (Maluszynski *et al.* 2003). Compared with other methods of doubled haploid formation, that is, gynogenesis and pollination with irradiated or alien pollen, microspore embryogenesis has become a better choice for haploid breeding in several economically interesting crops (Chupeau *et al.* 1998; Datta 2005).

Fundamentally, cassava (*M. esculenta*) is a crop able to grow on marginal lands. It has a considerable potential to eliminate hunger in the poor world, especially in Asia, Africa and Latin America. With the threat of climate change, this crop has been recognized as being highly resilient to future climatic changes (Jarvis *et al.* 2012). Apart from its culinary value, it plays a major role in the starch industry (Davis *et al.* 2003) since starch can easily be extracted from its roots because of their low levels of protein and fat (Munyikwa *et al.* 1997), thus reducing the processing cost. Furthermore, the quality of cassava starch enables its use in various industries (Zhao *et al.* 2011). With the increasing demand for renewable energy, cassava is one of the major crops that can be used to produce bioethanol with minimum processing cost. With these trends, increasing research is currently being carried out to improve this crop. Two natural cassava starch mutations have been reported recently: amylose-free waxy starch through inbreeding (Ceballos *et al.* 2007) and small-granule, high-amylose starch through induced mutation with γ-rays (Ceballos *et al.* 2008). Furthermore, cassava has been biofortified with additional nutrients such as β-carotene and vitamin A (La Frano *et al.* 2012), which is beneficial to people living in poverty in Asia, Africa and Latin America. However, crop improvement is hampered by the heterozygous nature of the crop. A stronger pipeline for the production of homozygous cassava lines would greatly enhance the number of new cassava varieties not only with further added nutritional value, pest and disease resistance, and high yield but also by improving the crop in different novel dimensions. Asparagus cassava is an example of a new form of cassava that was produced by inbreeding of cassava and which greatly increased planting density (Hernan Ceballos, pers. comm.).

Our laboratory recently published a reproducible method for microspore-derived callus production via anther culture (Perera *et al.* 2014). However, an efficient protocol for plant regeneration is yet to be developed. Evidence was provided for cassava microspores being able to enter the sporophytic pathway within the anther, and genetic tests established that the callus lines produced were of microspore origin. Earlier research on haploid induction from cassava microspores by anther culture (Moh 1975; Mukherjee 1996; Woodward and Puonti-Kaerlas 2001; Chirinos *et al.* 2006; Xi *et al.* 2009) has been inconclusive in terms of origin or ploidy.

Microspore reprogramming for triggering the sporophytic pathway from its normal gametophytic pathway is the first step in the establishment of a microspore culture technique. The most critical factors affecting this transition are the genotype and growth conditions of the donor plants, collection of buds in the optimal stage, stress treatments, as well as the composition of the culture medium and culture conditions (Perera *et al.* 2008; Irikova *et al.* 2011). Here, we report that the most responsive microspore developmental stages for reprogramming into sporogenic microspores are different in two cassava genotypes, that is, the tetrad stage and the free microspore stage. We also report the development of a novel medium for cassava microspore culture. Light and electron microscopic studies characterized the reprogramming process and allowed us to identify difficulties encountered in the reprogramming process.

## Methods

### Plant material and donor plant growth conditions

Two cassava genotypes, SM 1219-9 and TMS 60444 (hereafter designated as SM and TMS), were used to optimize the culture conditions for microspore culture as the first step in the production of doubled haploids. The donor plants were grown in three locations at the headquarters of the International Center for Tropical Agriculture. Year-round flower bud production was obtained by maintaining the plants with sprinkler irrigation and fertilizer application. Manual weed control and biological pest control were practised in order to maintain the plants with minimal chemical application.

Inflorescences were picked before 0900 h, and flower buds ranging from 2.0 to 2.8 mm in diameter were collected. The number of buds required was determined based on the calculation of 50 buds per 10 mL of medium on a culture plate (60 mm × 15 mm). The selected buds were disinfected under aseptic conditions with 70 % ethanol for 1 min, followed by a 10 % clorox solution containing 30 µL Tween-20 for 20 min in two changes. Then the buds were rinsed four times with sterile distilled water (Milli-Q Millipore, Billerica, MA, USA). The isolated buds were given a cold pretreatment at 10 °C for 3 days in the dark. After pretreatment, the buds were used for microspore isolation.

### Microspore isolation and culturing

Up to 400 buds were used for a single isolation of microspores. The buds were placed in a Waring commercial blender (Eberbach Corporation, Ann Arbor, MI, USA) together with a 10 % sucrose solution and were blended for 20 s at low speed. The resulting suspension was passed through filters made of nylon mesh (Spectrum^®^ Laboratories, Inc., Rancho Dominguez, CA, USA) with different pore sizes. Anther debris was removed by ﬁltering the blend through 149-µm mesh. The resulting suspension containing microspores and smaller debris was then filtered with 104-, 70- and 41-µm meshes sequentially and the cells remaining on each mesh were collected separately to different 15-mL falcon tubes with 10 mL of 10 % sucrose solution. Different sucrose concentrations (10, 20 or 30 %, either independently or by layering), centrifugation speeds (1000 and 500 rpm; Kubota, Tokyo, Japan) and times (0.5, 1 and 5 min) were tested to optimize the isolation and purification protocol. Then the isolated microspores were suspended in a 10 % sucrose solution or culture medium by adjusting the volume to be dispensed to a density of 5 × 102 per mL. Each Petri plate (60 × 15 mm) contained 10 mL of liquid culture medium. All the activities were performed in a laminar flow cabinet under aseptic conditions.

In a pilot study, the microspore response of the extracts of 70 and 41 was tested using five different culture protocols with different culture media, namely, modified MS developed for cassava anther culture (Perera *et al.* 2014), B/AT3 (as described by Touraev and Heberle-Bors 1999), NLNS preculture medium followed by the complete culture media NLN basal medium (as described by Kim *et al.* 2008), and NLN and NLNS medium supplemented with 10 % sucrose, 800 mg L^−1^ glutamine and 12 mg L^−1^ picloram. Microspores were cultured in the latter two media after applying 3 days of cold pretreatment at 10 °C whereas the relevant temperatures as described in the protocols were used for the first two protocols.

After adjusting the pH to 5.8, all media were sterilized by ﬁltration through 0.45-µm Nalgene^®^ disposable bottle-top filters (Thermo Scientific™ Nalgene™ Rapid-Flow). After filter sterilization, timentin (Sigma Aldrich, Lyon, France) was added at a concentration of 160 mg L^−1^ to control contamination. Culture plates were sealed with Paraﬁlm and incubated at 28 °C in the dark. The cultures were observed under an inverted microscope (Olympus IMT-313, Tokyo, Japan) and pictures were taken with a digital colour camera (MotiCam5 of 5.0, Richmond, Canada).

#### Effect of Fe and Cu ions on reprogramming

Two experiments were conducted to increase the frequency of reprogramming microspores using additional ion concentrations supplemented to the above-mentioned modified NLNS medium. In the first experiment, three concentrations of sodium ferrous ethylene-diamine-tetraacetic acid (NaFeEDTA), 45.88, 64.20 and 82.50 mg L^−1^, were compared with the normal concentration of 27.50 mg L^−1^ in the basal medium. The experiment was repeated six times. In the second experiment, CuSO_4_·5H_2_O, in concentrations of 0.255, 0.260, 0.265, 0.280 and 0.300 mg L^−1^, was tested over the concentration available in the basal medium, that is, 0.250 mg L^−1^. The experiment was repeated 10 times.

### Statistical analysis

Microscopic observations of the cultures were made at daily intervals during the first week and then at weekly intervals. The microspores with altered aspects, mainly enlargement, were considered as the reprogrammed ones. The average reprogramming rate of the cultured microspores was calculated as the number per hundred flower buds. Data presented were representative of a minimum of 2000 microspores observed per time point in at least six independent experiments as specified under each experiment. Mean values ± SD of the frequencies of reprogramming were calculated. The significance of the differences in mean values of the treatments in each experiment was tested using the statistical package SAS (SAS Institute 1999). Chi-square or maximum likelihood ANOVA was conducted using the PROC CatMod Procedures of PC/SAS. The treatment means were compared with the control treatment based on standard error, 95 % confidence intervals or orthogonal contrast coefficients (Compton 1994). All experiments were analysed as a completely randomized design.

### Microscopic evaluation

#### Microspore viability

Viability of the microspores in the different steps or time points was assessed by using fluorescein-diacetate (FDA) and Alexander staining. Fluorescein-diacetate-stained microspores were observed under an epi-fluorescence microscope (Ernst Leitz Wetzlar GmbH Leitz, Wetzlar, Germany; excitation 490–512 nm, emission 515–535 nm) whereas tetrads stained with Alexander staining were observed under the same microscope without fluorescence. Ten slides were prepared at each step and the counts were scored from three fields of each slide. Counts were made according to the number of viable microspores available in the tetrads and then the percentage was calculated over the total tetrads. At least a total of about 500 tetrads were counted at each step. The experiment was repeated two times.

#### Scanning electron microscopy

Four samples containing freshly collected, enlarged and non-responsive (unchanged) microspores in genotype SM and responsive microspores from TMS were examined. The samples were first fixed in 2.5 % glutaraldehyde (in 0.1 mol L^−1^ phosphate buffer, pH 7.2) overnight at 4 °C, and post-fixed in aqueous osmium tetroxide (1 %) for 1 h. The samples were then washed three times with distilled water and dehydrated in a graded ethanol series (25, 50, 75, 90 and 100 %, three times). Critical-point drying was performed in a Samdri-78-A (Tousims Research Corporation, Rockville, MD, USA). The dried specimens were mounted onto aluminium cylinders, and gold was sputtered in a Hummer VII sputtering system (Anatech Ltd, Union City, CA, USA). Samples were observed with a scanning electron microscope (JEOL JSM820, Tokyo, Japan). Images were captured using a Nikon D3000 in a Taylor adaptation system (M.E. Taylor Engineering Inc., MD, USA). More than a thousand tetrads and microspores were screened in each sample.

#### 4′,6-Diamidino-2-phenylindole staining of nuclei

The nuclear status of cultured tetrads and microspores of genotypes TMS and SM was assessed by 4′,6-diamidino-2-phenylindole (DAPI, Sigma Aldrich) staining. Cultures and fresh samples were transferred to an Eppendorf tube and the culture medium was removed after centrifugation. The pelleted microspores were then fixed in Farmer's solution (ethanol : glacial acetic acid = 3 : 1, v/v) overnight at 4 °C. The fixative was decanted after centrifugation and washed three times in phosphate-buffered saline (PBS) buffer (1×; pH 7.4) by centrifugation at 500 rpm for 1 min. The pellet was suspended in DAPI (100 ng mL^−1^) in 1× PBS buffer and incubated for 10 min in the dark. The exine of microspores of SM was digested according to Wang *et al.* (2011) prior to the DAPI staining procedure. Observations were made using light and epi-fluorescent microscopy (Ernst Leitz Wetzlar GmbH Leitz) with 340 nm excitation and 488 nm emission.

#### Histological analysis

For histological analyses, microspores isolated from 50 buds in each of the following conditions were evaluated. Fresh microspores isolated just after collecting the buds and after application of cold pretreatment for 3 days at 10 °C were sampled. After inoculating the isolated tetrads or microspores in modified NLNS medium, sampling was performed at 1-, 3-, 5- and 7-day intervals. Another analysis was conducted with genotype SM to test whether there was any correlation between enlargement and reprogramming of the microspore. From the cultures, enlarged microspores >104 µm in diameter and in the range of 104–70 µm were sampled 1 week after culture initiation. Samples were fixed overnight in FAA (50 % ethanol : 10 % formaldehyde : glacial acetic acid = 18 : 1 : 1) and then dehydrated through a graded ethanol series (10, 20, 30, 50, 70, 80, 90, 95 and 100 %, v/v) and 100 % butanol for 30 min, changing each solution twice at 15-min intervals. After fixation, samples were embedded in Technovit 7100^®^ resin (Heraeus Kulzer GmbH, Wehrheim, Germany). Sections of 3-μm thickness were obtained using a rotary microtome (Histostat; Reichert Scientific Instruments, Buffalo, NY, USA), and five slides were prepared for each sample. Slides were stained with periodic acid Schiff's reagent (Sigma Aldrich) for starch (pink in colour) and naphthol blue black for proteins (blue in colour). All sections were studied under a light microscope (DM500; Leica GmbH, Wetzlar, Germany) and photographed with an attached camera (ICC50HD; Leica GmbH).

#### Transmission electron microscopy

Four samples containing freshly collected, enlarged and non-responsive (unchanged) microspores in SM and responsive cultures from TMS were examined. The microspores were fixed in 2.5 % glutaraldehyde (in 0.1 mol L^−1^ phosphate buffer, pH 7.2) overnight at 4 °C and post-fixed in aqueous osmium tetroxide (1 %) for 1 h. After three washings with distilled water, the microspores were embedded in 1 % (w/v) agar for easy handling. Dehydration was performed in 25, 50 and 75 % ethanol for 20 min. Specimens were immersed in 2 % uranyl acetate (in 75 % ethanol) for 12 h at room temperature, followed by further dehydration in 90 % (1 bath) and 100 % ethanol (3 baths) and acetone (3 baths) for 20 min. After fixing the microspores with acetone-Spurr epoxy resin (DER^®^ 736; Ted Pella Inc., Redding, CA, USA) (1 : 1) followed by pure resin for 1 h (3 changes, Spurr 1969), samples were embedded in resin polymerizing at 65 °C for 16 h. Ultra-thin sections obtained with the PowerTome X ultra-microtome (RMC, Tucson, AZ, USA) with a diamond knife at 70 nm were stained with uranyl acetate and lead citrate (Reynolds 1963). The sections were examined with a transmission electron microscope (JEOL JM1010), and the images were captured and analysed using AnalySIS 3.0 software (Soft Imaging System GmbH, Münster, Germany).

## Results

### Testing of the basal media composition and culture procedure

Among the tested culture media, a positive response was observed only in the modified NLNS medium supplemented with 10 % sucrose, 800 mg L^−1^ glutamine and 12 mg L^−1^ picloram. Cultured microspores remained unresponsive in all the other media tested. Thus, the experiments were continued with the modified NLNS medium.

### Induction of microspore reprogramming

**Figure 1.**
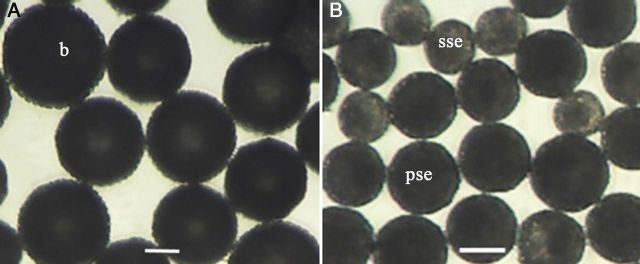
Morphological aspects of isolated microspores from genotype SM1219-9 of *M. esculenta*. (A) The suspensions consisted of microspores of black (b) colour having a well-developed exine captured in the 70– to 104-µm extract. (B) Microspores with partially synthesized exine (pse) of less intense black and slightly synthesized exine (sse) in yellowish brown in the 41– to 70-µm extract.

Freshly isolated microspores were separated into three size groups by filtering. In both genotypes, the tested groups of microspores were in the size ranges of 149–104, 104–70 and 70–41 µm, designated hereafter as 104, 70 and 41 extracts. An almost-clear debris-free extract of each size category was obtained by centrifuging the suspensions with 10 % followed by 30 % sucrose solution independently at different speeds: 104 and 70 extracts at 500 rpm and the 41 extract at 1000 rpm, each for 1 min. In the 104 and 70 extracts, the suspensions consisted of microspores of different sizes of black colour when viewed under an inverted microscope (Fig. 1A) with a well-developed exine, whereas in the 41–70 µm extract the microspores contained a partially synthesized exine of less intense black colour and a slightly synthesized exine of yellowish brown colour (Fig. 1B). Tetrads, mixed with free microspores in the mid or late uni-nucleate stage, were captured by the 70 extract.

Morphological changes were observed in the cultured microspores only in extract 70, whereas the 104 and 41 extracts remained unchanged without any observable response, even though the cultures were maintained for as long as 4 months. Different reprogramming responses were obtained from the 70 extract in the two genotypes tested: a significant enlargement of the microspores in tetrads in TMS, and enlargement of free microspores (the diameter being >104 µm, the upper limit of the diameter of this group of extract) in SM. Even though enlarged free microspores in TMS and enlarged microspores of tetrads in SM were observed, their frequencies were extremely low. Therefore, any size difference occurring in any of the four microspores in a tetrad in TMS and the enlarged microspores in SM were considered as positive responses.

**Figure 2.**
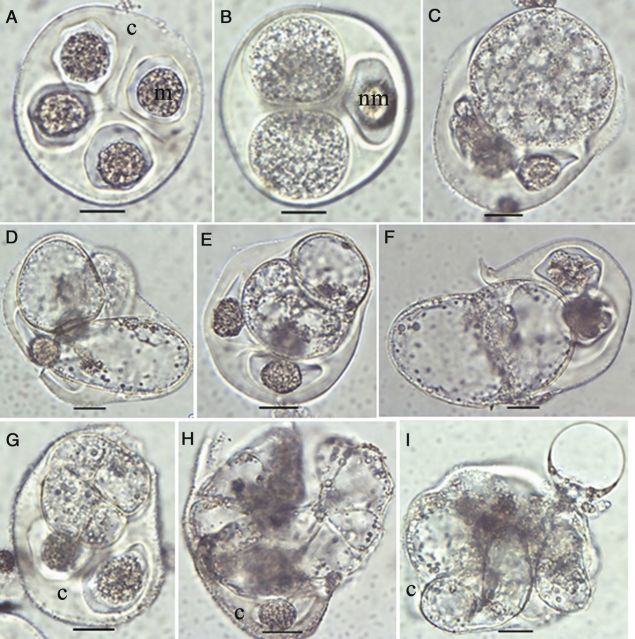
Morphological aspects of microspore reprogramming in tetrads of *M. esculenta* genotype TMS 60444. (A) An immature tetrad containing round-shaped microspores (m) within the callose (c) wall. (B) A tetrad containing two enlarged microspores. Note that the non-responding microspore (nm) remained unchanged. (C) Small vacuole formation upon further enlargement in the responsive microspore. (D) Formation of a large vacuole. (E) A divided small microspore. (F) A divided enlarged microspore. Note that the dividing structure has come out from the callose wall. (G) A four-celled microspore. (H) An eight-celled microspore. (I) Multi-celled structures found in the cultures (A: fresh; B: 2 days; C: 3 days; D: 5 days; E–I: 7 days after culture initiation of the tetrad; scale bar = 20 µm).

In the cultured tetrads of TMS, the four microspores were morphologically similar in size and shape (Fig. 2A). The first sign of an enlargement of the microspores in tetrads was observed 2 days after culture initiation (Fig. 2B). These microspores enlarged further and formed small vacuoles (Fig. 2C) 3 days after culture initiation, and then the cytoplasm turned into a lighter state (Fig. 2D). By fusion of the small vacuoles 5 days after culture initiation, large vacuoles formed, and the cells acquired oval or oblong shapes (Fig. 2D). At this time, cytoplasmic streaming could be observed in the cells. The tetrads containing at least one enlarged microspore (remaining enclosed or exposed but still attached to the callose wall) were counted as responsive individuals. Seven days after culture initiation, structures with two (Fig. 2E and F), four (Fig. 2G) and eight cells (Fig. 2H) and then multi-cellular structures (MCSs) (Fig. 2I) were observed. The non-responsive microspores of the responsive tetrads remained unchanged whereas the responsive ones either grew larger or divided further. In most cases, only one microspore of the tetrad was reprogrammed, whereas two or three cells were responsive only in rare cases (as in Fig. 2D). The non-responsive microspores in the responsive tetrads and all the microspores in the non-responsive tetrads contained highly dense cytoplasm and, in some cases, escape of cytoplasm out of the cells occurred, indicating cell death. Among the cultured microspores and tetrads captured in the 70 extract, meiocytes were found, which also enlarged and then divided (data not shown).

**Figure 3.**
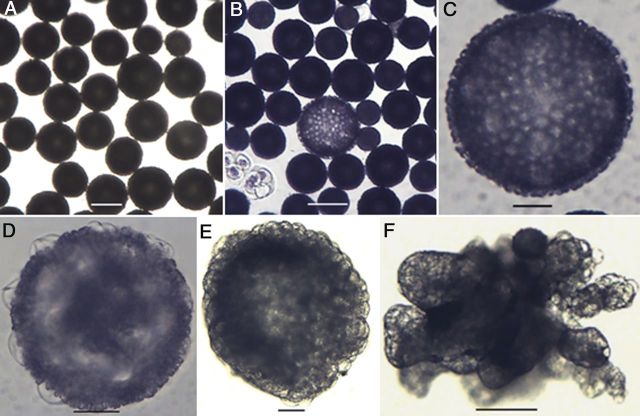
Morphological aspects of microspores of *M. esculenta* genotype SM 1219-9. (A) Cultured fresh microspores. Note the different maturity stages represented by different sizes. (B) A reprogrammed microspore showing significant enlargement over non-responsive ones. (C) Close-up of an enlarged whole gametophyte. (D–E) MCS developing through the aperture area. (E) A globular MCS formed in the subculture medium containing 6-benzylaminopurine. (F) A micro-callus formed after subculturing into 2,4-d-containing medium (A: fresh; B–D: 7 days after; E–F: 15 days after culture initiation; scale bars (A, B, F) = 100 µm, (C–E) = 30 µm).

In genotype SM, cellular changes occurring within the free microspores could not be observed due to the thick exine. The non-responsive microspores remain unchanged in size or appearance (Fig. 3A), whereas enlargement was the only morphological marker of induction (Fig. 3B) to be seen 3 days after culture initiation. Microspores were considered as enlarged only if the diameter was >104 µm, the upper limit of the extract range. The intensity of the dark-coloured exine decreased in these enlarged microspores (Fig. 3B and C). After about 1 month, some transparent protrusions were observed in the enlarged microspores and these protrusions were concentrated in one part of the microspore (Fig. 3D). Globular MCSs (Fig. 3E) or micro-calli (Fig. 3F) were present in the cultures about one and a half months after culture initiation.

#### Effect of Fe and Cu on reprogramming

**Figure 4.**
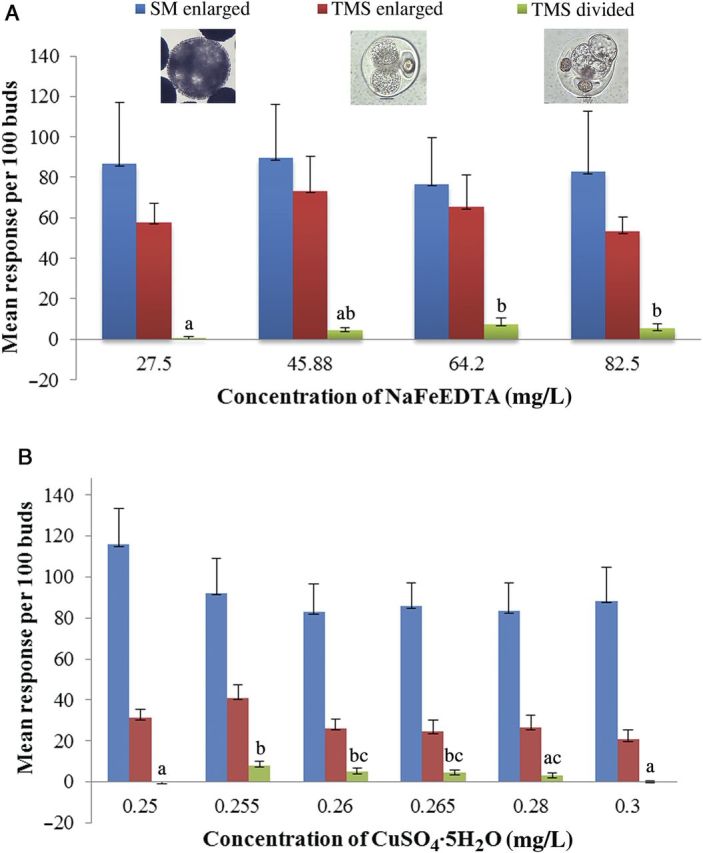
Response of cultured microspores of *M. esculenta*. (A) Effect of NaFeEDTA. (B) Effect of CuSO_4_·5H_2_O. In the cultures of TMS 60444, the tetrads containing enlarged and divided microspores were counted whereas in SM 1219-9 only the enlarged microspores were counted. Values followed by the same letters are not significantly different.

This experiment revealed that additional NaFeEDTA resulted in an increase in the frequency of MCSs in TMS over the control of the concentration in the basal medium, in which only enlargement occurred. Irrespective of the concentration of NaFeEDTA, enlargement was observed in both the microspores in tetrads of TMS and microspores of SM, having an average frequency of 63 and 84 per 100 buds (Fig. 4A). The concentration of 64.20 mg L^−1^ NaFeEDTA added to the medium showed a significant increase in the divided microspores enclosed in tetrads with 8.3 per 100 buds (*P* < 0.05; LSD = 5.2), whereas no division was observed in the medium with a normal level. A similar observation was made in the second experiment, in which the effect of CuSO_4_·5H_2_O significantly increased the frequency of divided microspores of tetrads in TMS at a concentration of 0.255 mg L^−1^ (an additional 0.005 mg L^−1^ over the control; *P* ≤ 0.001; LSD = 3.7), whereas enlargement occurred irrespective of the concentration added to the culture medium (Fig. 4B). The medium supplemented with CuSO_4_·5H_2_O gave rise to an average of 28 enlarged microspores in tetrads and 91 enlarged microspores per 100 buds in TMS and SM, respectively. Higher concentrations reduced the frequency of dividing microspores.

### Microscopic analysis of the cultured microspores

#### Viability of the microspores in tetrads

**Figure 5.**
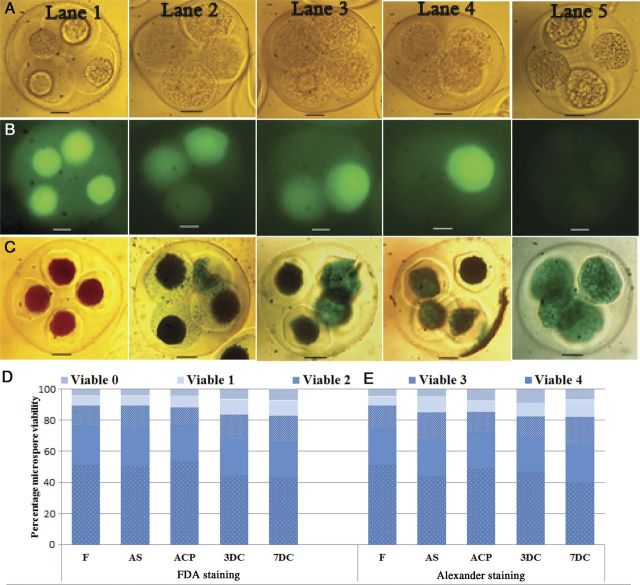
Quantitative and qualitative demonstration of the microspore viability of tetrads of *M. esculenta* genotype TMS 60444. Fluorescein-diacetate-stained microspores of tetrads under (A) a light microscope and (B) a fluorescent microscope. (C) Microspores of tetrads stained with Alexander stain. Lane 1: tetrads with four viable microspores; Lane 2: with three viable microspores; Lane 3: with two viable microspores; Lane 4: with one viable microspore; Lane 5: without any viable microspores in the tetrads (scale bars = 20 µm). (D and E) Quantitative data of microspore viability in tetrads after sterilization (AS), 3 days after cold pretreatment (ACP), 3 days after culture (3DC) and 7 days after culture (7DC) as compared with fresh (F) tetrads with FDA (D) and Alexander staining (E).

In order to find the reason for the low response in TMS, first the viability of the microspores in tetrads was evaluated by FDA and Alexander staining. The viability of the microspores in tetrads at the time of bud collection was compared with the viability at the different steps of culture protocol. Either all four microspores (Fig. 5, Lane 1) or three (Fig. 5, Lane 2), two (Fig. 5, Lane 3) or one microspore (Fig. 5, Lane 4) were viable in the tetrads when tested with FDA (Fig. 5A–C) in frequencies of 51.3, 25.2, 13.1 and 6 % in the freshly collected buds, whereas the frequency of tetrads with all four non-viable microspores (Fig. 5, Lane 5) was 4.4 % (Fig. 5D). The viability of the microspores did not change significantly during the culture process. Similar results were obtained with the Alexander staining (Fig. 5C) and the frequency of viability resulting from FDA was comparable with that of Alexander staining (Fig. 5E).

#### Scanning electron microscopy

**Figure 6.**
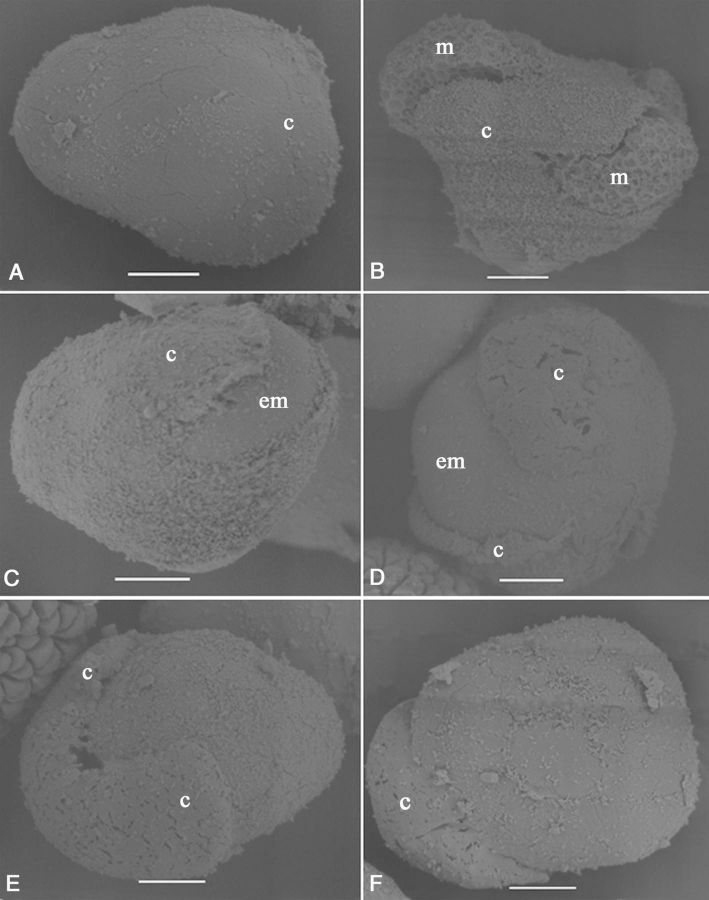
Scanning electron micrographs showing aspects of cultured tetrads of *M. esculenta* genotype TMS 60444. (A) Fresh tetrad. (B) Microspores (m) coming out of the callose (c) due to centrifugation pressure. Note the exine wall present on the surface of the microspores. (C) The enlarged (em) or divided microspore coming out of the broken callose wall. (D) Expanded callose wall opening with further enlargement of the microspore. (E and F) The callose wall is limited to only one part while the structure develops (scale bar = 10 µm).

To study the surface structures of responsive and non-responsive microspores in more detail, scanning electron microscopy (SEM) was used. The surface of the callose matrix containing the four microspores in TMS was smooth (Fig. 6A). The surface of the microspores could not be observed when they were enclosed within the callose matrix. However, microspores that were emerging through the callose wall (broken in centrifugation during the extraction process) had a rough surface with ridges (Fig. 6B), indicating the existence of an exine. In contrast, microspores that broke through the callose wall in culture had a smooth surface (Fig. 6C). Larger or divided microspores produced a wider rupture site in the callose wall that was limited to one corner of the tetrad (Fig. 6D–F). The dividing microspores could be identified by the inward movement of the surface of the structure (Fig. 6D–F).

**Figure 7.**
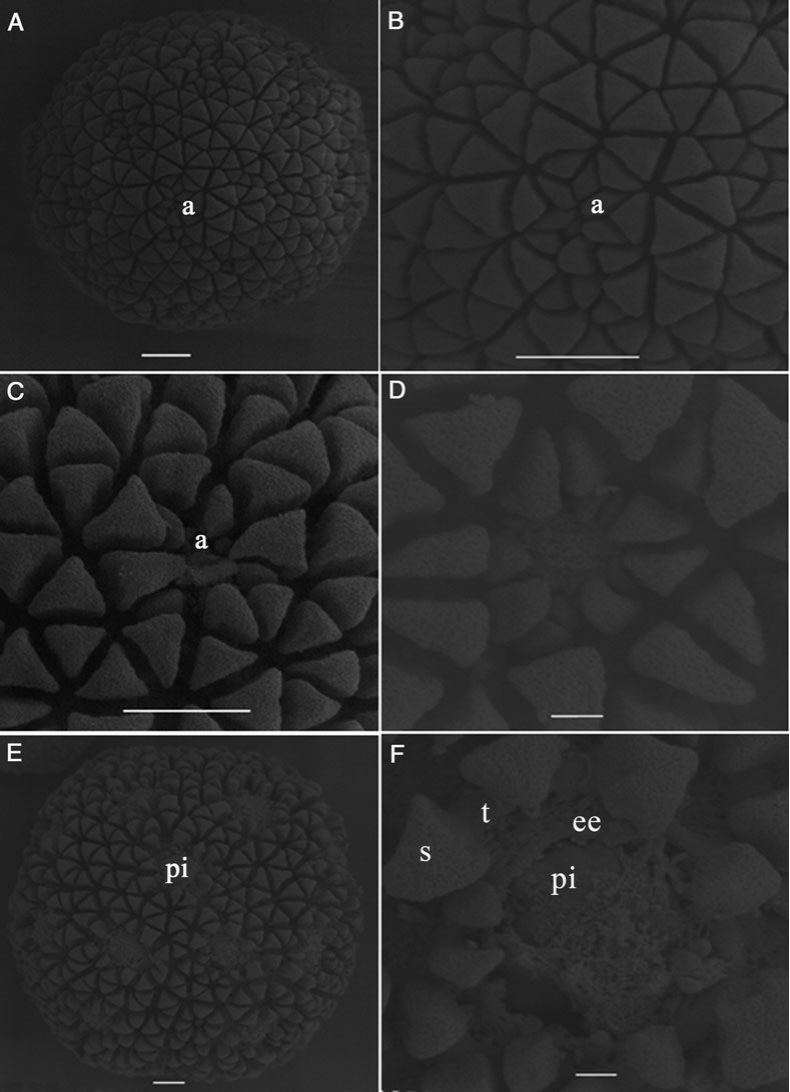
Scanning electron micrographs showing aspects of cultured tetrads of *M. esculenta* genotype SM 1219-9. (A) A fresh microspore. Note the differential size and shape of sculptured particles at the aperture (a) area as compared with the rest (scale bar = 10 µm). (B) Close-up view of the pattern of exine sculptures at the aperture of a fresh microspore (scale bar = 10 µm). (C and D) Changes occurring at the aperture in the enlarging microspore. Note that the aperture is opening up and the intine becomes visible (scale bars (C) = 10 µm and (D) = 2 µm). (E) A microspore with protruded intine (pi) through the aperture (scale bar = 10 µm). (F) Close-up view of an aperture where the exine particles are much expanded, the aperture has opened well and where the intine is coming out, making a protrusion. Note the different layers of the exine: end exine (ee), tectum (t) and the sculpture particles (s) located on the tectum (scale bar = 2 µm).

Immature microspores at the mid to late uni-nucleate stage of genotype SM, which were captured in the 70 extract, had exine sculpture particles on their surface, present in an organized manner and forming the typical cassava exine. The exine formed in a regular geometric pattern by the deposition of triangular sculpture elements while the numerous apertures were indicated by the presence of concentrated small sculpture particles (Fig. 7A). The microspores had a fenestrate pore arrangement and were therefore poly-aperturate. The elements located at the aperture were smaller than the rest (Fig. 7B–D). During microspore enlargement, the exine showed interesting changes. With the enlargement of the microspores, the distance between the particles increased, and the intine underlying the exine was clearly visible at the aperture (Fig. 7D). Upon further enlargement, protrusions could be observed at the pores (Fig. 7E). The sculpture elements on the tectum and the absence of a tectum in the pore area were clearly observed when further enlargement of the microspore occurred (Fig. 7F). The three layers of the exine, endexine, columellae and tectum could be identified clearly, as well as the intine. The intine pushed through the pore, producing an oncus on the surface.

#### DAPI staining of nuclei

**Figure 8.**
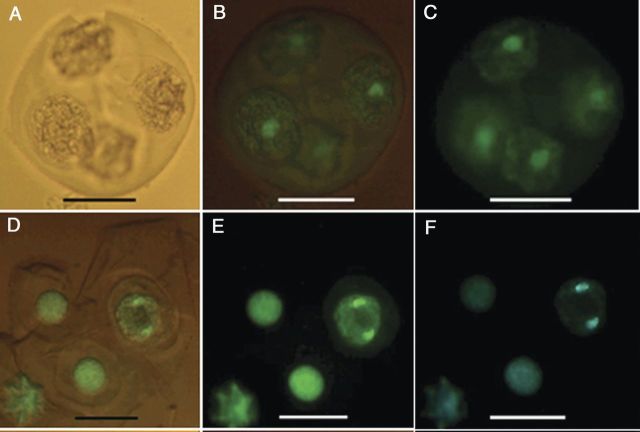
Fluorescence micrographs of nuclei in cultured tetrads of *M. esculenta* genotype TMS 60444. (A–C) A fresh tetrad without fluorescence and with partial and full fluorescence under a green filter. (D–F) Cultured tetrad containing an enlarged microspore undergoing mitosis under partial and full fluorescence with a green filter and full fluorescence with a blue filter (scale bars = 22 µm).

**Figure 9.**
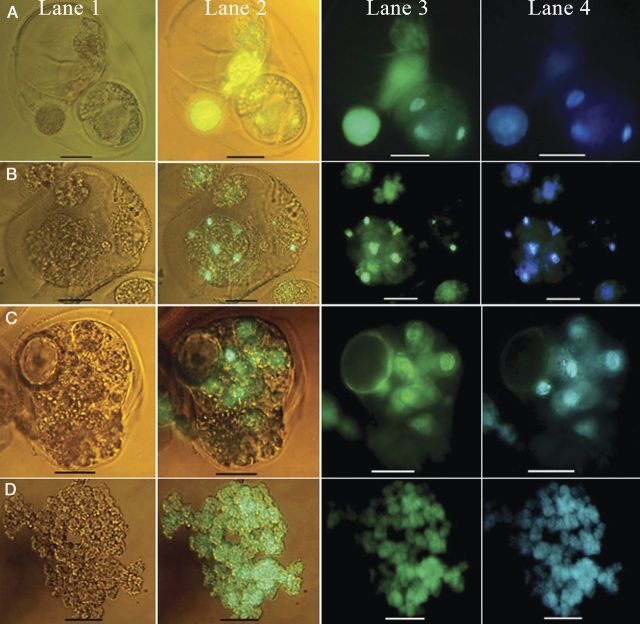
Fluorescence micrographs with aspects of nuclear division in the cultured tetrads of *M. esculenta* genotype TMS 60444. (A) Four-celled tetrad with three nuclei visible. (B) Eight-celled tetrad with six nuclei visible. (C) Multi-nucleate structures 7 days after culture. (D) Callus 1 month after culture. Lane 1: without fluorescence; Lanes 2 and 3: with partial and full fluorescence with a green filter, respectively; Lane 4: full fluorescence with a blue filter (scale bars = 22 µm).

The nuclear status of the responding and non-responding microspores of tetrads in TMS was studied by staining the nuclei with DAPI. The degree of chromatin condensation in the nucleus was used as a marker to distinguish the status of the nuclei in the cell cycle. Fresh and non-responsive microspores in tetrads contained a single prominent nucleus (Fig. 8A). The study revealed cells with different nuclear status (as shown in Fig. 8B) even though they did not show any remarkable cell enlargement and were considered as non-responsive tetrads by visual observation. In microspores that did not show a significant enlargement, a nuclear division (Fig. 8B) was observed, indicating that reprogramming was occurring even without a visual sign. These results clearly demonstrated that visual counts did not reflect actual reprogramming frequency, which should actually be higher than that present in Fig. 4. However, confirming the visual observation of division in microspores of tetrads, the structures with four (Fig. 9A), eight (Fig. 9B) and multiple (Fig. 9C) cells with prominent nuclei were clearly demonstrated by DAPI staining. Furthermore, MCSs derived from the tetrad microspores showed numerous stained nuclei (Fig. 9D). However, in some cases, it was difficult to recognize whether the structures were multi-cellular or multi-nuclear because cell walls are difficult to see. In genotype SM, unfortunately, auto-fluorescence of the exine of the microspores prevented visualization of the nucleus despite the exine-digestion procedure.

#### Histological analysis

**Figure 10.**
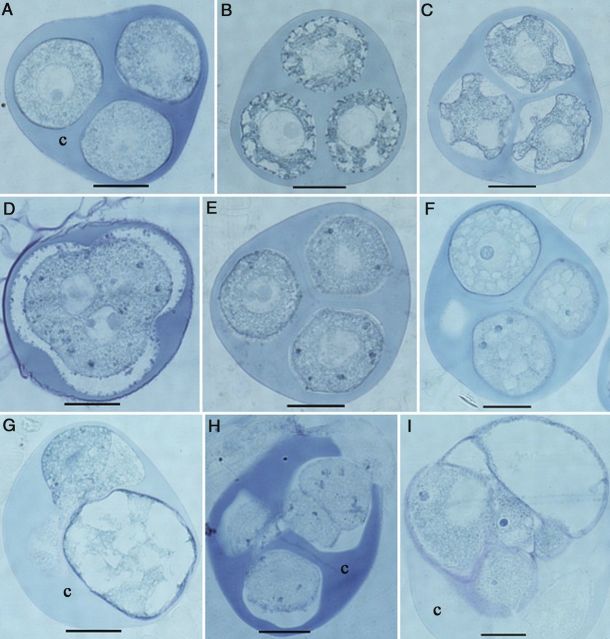
Histological aspects of the microspore reprogramming of *M. esculenta* genotype TMS 60444. (A–C) Fresh tetrads. (A) Immature tetrad microspores with smooth surface. (B) Tetrad microspore with initial deposition of exine particles and initial inward movement of cytoplasm. (C) Mature tetrad microspores with deep folds forming on the irregular surface of the microspore. (D) A meiocyte among the cultured tetrads. Note that cytokinesis has not yet occurred even after meiotic karyokineses. (E) Significant appearance of protein deposits after applying 3 days of cold pretreatment (blue particles). (F) Small vacuoles in microspores 1 day after culture. (G) Enlarged microspore with large vacuoles 3 days after culture. (H) Cell division in microspores 3 days after culture. (I) A multi-cellular structure still enclosed in the callose wall (c) 7 days after culture (scale bars = 20 µm).

The cellular changes occurring during the transition from gametophytic to sporophytic development after culture initiation were also studied by sectioning. Among the fresh tetrads of TMS, different stages with specific characteristic features could be distinguished. Round-shaped microspores with a uniform cytoplasm had a smooth surface (Fig. 10A); oval-shaped microspores with a highly dense cytoplasm had initial exine particles on the surface, probably consisting of sporopollenin (Fig. 10B), confirming the scanning electron microscopical findings. Irregular-shaped microspores had a deeply folded surface with more exine deposits (Fig. 10C). In the first two stages, the nucleus was larger than in the latter. Meiocytes in various stages of meiosis were also observed (Fig. 10D) — interestingly, the stage that the four tetrad nuclei were initially not surrounded by a cell wall. The first change in all meiocytes (Fig. 10D) or microspores (Fig. 10E) of a tetrad occurred 3 days after cold pretreatment. Dark blue granules appeared in the cytoplasm indicating the presence of proteinous structures stained by naphthol blue black (Fig. 10D and E). These spots were continuously present throughout the culture period in non-responsive microspores, whereas they disappeared in the responsive ones when they enlarged. Two additional features could be identified based on the status of the cytoplasm in the microspores. In certain microspores in tetrads, numerous small vacuoles formed 1 day after culture initiation (Fig. 10F) and then large vacuoles formed during culture, apparently by fusion (Fig. 10G). Certain microspores containing a uniform cytoplasm divided 1 day after culture initiation (Fig. 10H) and developed into an MCS within 1 week after culture initiation.

**Figure 11.**
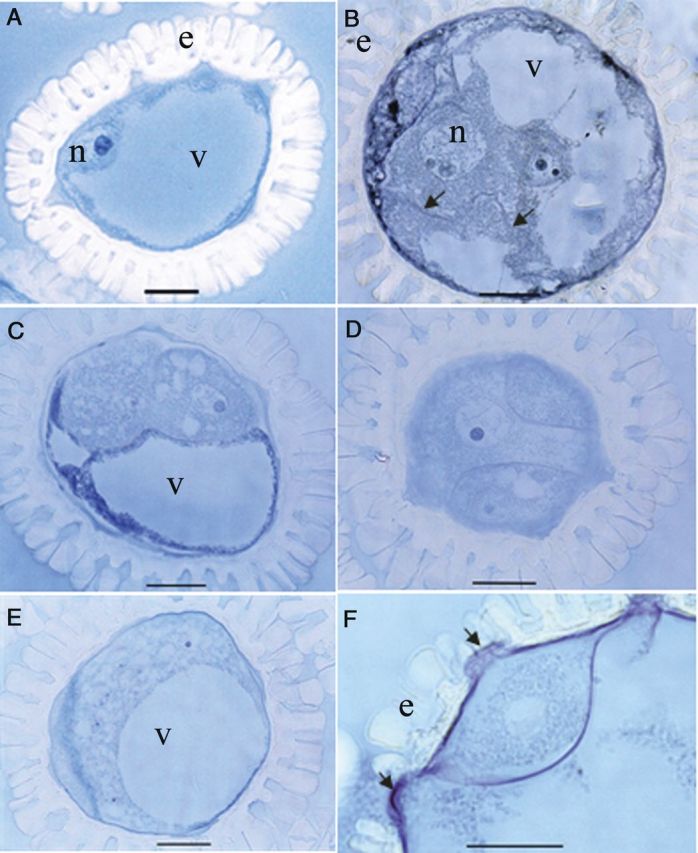
Histological aspects of microspore reprogramming of *M. esculenta* genotype SM 1219-9. (A) A fresh microspore at the late uni-nucleate stage with a prominent nucleus (n). Note the large vacuole (v) and the well-developed exine (e). (B) A multi-nucleate microspore after 1 day in culture. Note the developing cell wall (arrowhead) in the cytoplasm. (C) Two cells in a cultured microspore pushing the vacuole to one side. (D) Three cells in a cultured microspore present in the section away from the diameter of the microspore. (E) Multi-nucleate microspore in culture containing numerous nuclei in the cytoplasm. Note that the expanding cytoplasm pushes the vacuole to one side. (F) Magnified view of the microspore in (E) showing that the intine is pushing through the aperture black arrow (scale bars = 20 µm).

About 90 % of the microspores from the 70 extract of SM had a well-developed single vacuole and the nucleus in a peripheral position, indicating the late uni-nucleate stage prior to first pollen mitosis (Fig. 11A), whereas the rest had two to four vacuoles and a central nucleus, indicating the mid uni-nucleate stage. Even though no special change could be observed at the cellular level after cold pretreatment, three types of microspores were observed. In one type, nuclear division as well as cytokinesis were observed 1 day after culture initiation (Fig. 11B) and thereafter MCSs formed (Fig. 11C and D). The division was symmetrical (Fig. 11B), rather than asymmetrical as in regular, gametophytic first pollen mitosis. In the other type, numerous nuclei were present in the cytoplasm (Fig. 11E). In both multi-cellular and multi-nuclear structures, the divisions occurred in the non-vacuolated area of the pollen grain, filling the vacuolated area gradually with cells throughout the culture period (Fig. 11B, C and E). All these features were observed only in the enlarged microspores (>104 µm). As a common feature, the intine of the enlarged microspores was pushed out through the aperture, forming a protrusion through the aperture of the exine (Fig. 11F). This is consistent with the results from SEM (see below). The third type of microspore was small, that is, unchanged in size, and had one nucleus. Apparently, these were microspores that had not undergone reprogramming, but also did not pass through first pollen mitosis.

#### Transmission electron microscopy

**Figure 12.**
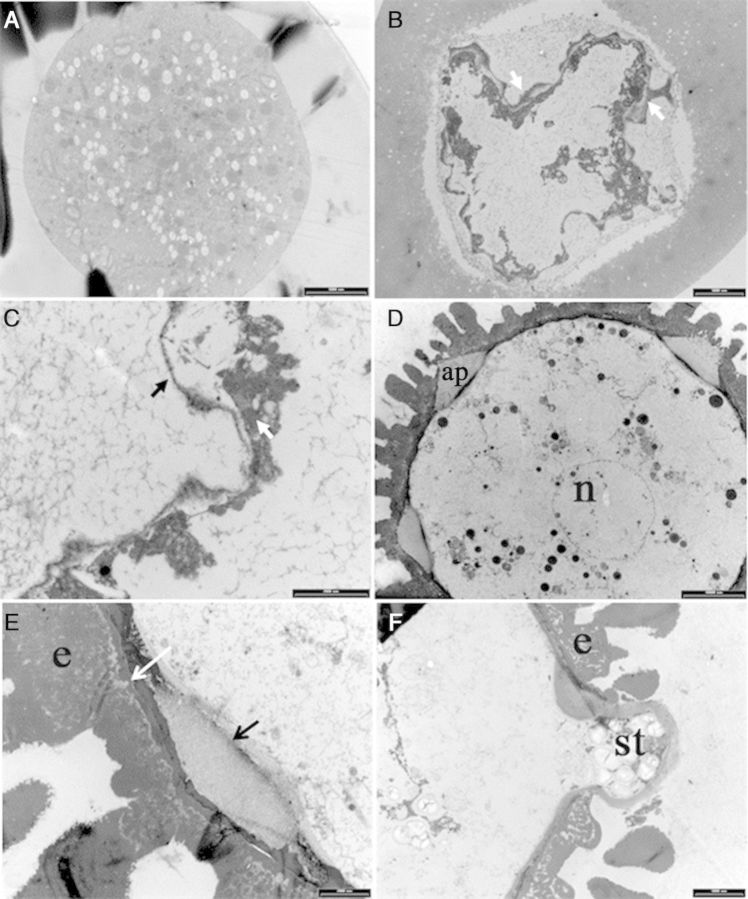
Transmission electron micrographs showing ultra-structural features of fresh and cultured tetrads of genotype TMS 60444 (A–C) and microspores of SM 1219-9 (D–F) of *M. esculenta*. (A) Round-shaped immature microspores enclosed in the callose of a fresh tetrad. Note the smooth surface and even cytoplasm (scale bar = 5000 nm). (B) Microspore with an undulating periphery enclosed in the callose of tetrads (scale bar = 5000 nm). Note the initial deposition of the exine material (white arrow). (C) Microspore in a fresh tetrad at higher magnification showing intine (black arrow) and exine (white arrow) (scale bar = 2000 nm). (D) Microspore with nucleus (n) and the aperture (ap) (scale bar = 10 000 nm). (E) Higher-magnification view of the exine (e) of a fresh microspore clearly showing the end exine (white arrow) and the intine (black arrow, scale bar = 2000 nm). (F) Cultured microspore 7 days after culture initiation (scale bar = 5000 nm). Note the intine protruding through the aperture and the starch (st) granules accumulated in the protrusion.

Fresh and cultured tetrads and microspores were further studied to confirm the observations made by classical histology. In TMS, the round-shaped microspores in tetrads with a smooth surface had only an intine (Fig. 12A) whereas microspores with a deeply folded surface showed exine particles at the periphery, which were deposited in a rather disorganized manner (Fig. 12C). Round-shaped microspores contained more organelles than microspores with a deeply folded surface. In SM, the fresh microspores were at the uni-nucleate stage (Fig. 12D). The exine at the aperture was thinner than around the rest of the microspore. An oncus was formed at the aperture by thickening of the intine pushing the cytoplasm with the cell membrane deeply inside the microspore (Fig. 12E). This was a characteristic feature of all uni-nucleate microspores. Enlarged microspores had a much thinner exine (Fig. 12F) than fresh ones or those that remained at the same stage in culture. In the enlarged microspores, cytoplasmic protrusions were found to extend through the aperture covered by the intine (Fig. 12F). This observation confirms the observations made by SEM and classical histology of enlarged microspores. In most cases, starch particles were seen in the protruding cytoplasm. The surface of these protrusions was totally free of exine material.

## Discussion

Developmental pathways of cultured microspores have been reported to vary depending on the plant species and in response to various stress treatments and microspore developmental stages (Sunderland *et al.* 1979; Raghavan 1986; Kim *et al.* 2004). There is a time window during microspore development in which microspores can be deviated effectively from their normal gametophytic programme for embryogenesis, representing a key for developing a protocol for microspore culture (Seguí-Simarro and Nuez 2005). The most common responsive stage is the uni-nucleate stage (Heberle-Bors 1989) in monocot species such as coconut (*Cocos nucifera*) (Perera *et al.* 2008) and barley (*Hordeum vulgare*) (Maraschin *et al.* 2005) and dicot species such as pepper (*Capsicum annuum*) (Kim *et al.* 2008; Lantos *et al.* 2009); however, the early bi-nucleate stage may also be responsive. Calli originating from meiocytes have been reported in tomatoes (*Solanum lycopersicum*) (Shtereva *et al.* 1998; Seguí-Simarro and Nuez 2005). Here, we report the second case of the induction of microspore embryogenesis from microspores in the tetrad stage, providing the first detailed analysis of reprogramming occurring in isolated cassava microspores.

Perera *et al.* (2013) correlated the size (diameter) of the gametophytes with the stages of male gametogenesis in SM 1219-9 and TMS 60444, and, based on this finding, flower buds were selected (ranging in diameter from 2.0 to 2.8 mm) containing developmental stages from tetrads via early, mid or late uni-nucleate stages to immature bi-nucleate and mature microspores. Since the microspores were separated into three size groups by filtering, each extract represented a range of developmental stages. In both SM and TMS, suspensions obtained by filtering with mesh sizes of 149–104 µm contained mainly late uni-nucleate microspores and more advanced stages (104 extract) while suspensions obtained with mesh sizes of 104–70 µm contained early to late uni-nucleate microspores and tetrads (70 extract), and suspensions obtained with mesh sizes of 70–41 µm contained early uni-nucleate microspores freed from the callose matrix of the tetrads (41 extract). Responsive microspores were present only in the 70-µm extract, consisting of microspores in the mid or late uni-nucleate stage and the tetrad stage. Furthermore, a genotypic effect was found, with microspores in the mid or late uni-nucleate stage being responsive only in the SM genotype, while tetrads were responsive in the TMS genotype.

The latter finding prompted us to study tetrad formation in more detail. After meiosis but still within the callose wall, cassava microspores passed through different stages with specific features. Initially, the microspores had a round shape and then became oval followed by further growth of the cell wall (plasma membrane and intine), which folded deeply into the cytoplasm. In addition, the nucleus in these tetrad microspores became smaller (Fig. 10C). This seems to be a specific property of cassava meiosis. Exine synthesis started when the microspores were still enclosed by the callose wall (see Figs 6B, 10B and C, 12B and C) and continued after release from the callose wall as shown in Fig. 1. On the other hand, enlarged microspores in cultured tetrads had a smooth surface without any exine particles attached to them. They may have originated from the round-shaped immature microspores with a smooth surface (as in Fig. 10A). Fig. 2 shows that the non-enlarged microspores in the responsive tetrads are round shaped with a smooth surface, strongly suggesting that the first of the three stages of microspore development within the tetrads is the critical stage for reprogramming. Thus, it can be hypothesized that the reason for the low response in microspore cultures of the 70 extract is the low frequency of microspores in this critical stage. Another reason for the low microspore response is the low viability of the tetrad microspores. Viability may be lost during isolation and culturing. Yet, from the results presented, it is clear that microspore viability in tetrads is quite high at the time of bud collection and that there is no significant loss of viability during microspore culture. However, the study clearly demonstrated that the number of tetrads with non-viable microspores (one, two or three among the four microspores in tetrads) is about 45 % (Fig. 5). This observation sufficiently explains why only one or two microspores are responsive among the four in tetrads (Fig. 2). A further finding related to reprogramming was the lack of an exine on the responsive microspores. Thus, these microspores may be responsive only before the gametophytic programme has started. A possible link between the presence of exine particles and the viability of the microspores in the same tetrads was not analysed. Thus, both hypotheses may influence microspore reprogramming in the cultured tetrads of the TMS genotype. It is therefore necessary to continue the studies in order to find out whether there are any slight changes in the developmental pattern of the microspores in the same tetrads.

Enlargement of microspores is considered a marker for androgenesis induction in many crops (Maraschin *et al.* 2005). Perera *et al.* (2014) reported that microspore enlargement is the first sign of entering into androgenesis in cassava, whereas no size change was observed in some microspores in the cultured anthers of SM 1219-9. However, the exact reason for enlargement was not demonstrated there. The thick and auto-fluorescent exine of cassava microspores made it impossible to detect changes occurring within the microspores by light and fluorescent microscopy. Clorox that was used to remove the exine (Wang *et al.* 2011) did not do so completely; thus, exine auto-fluorescence still interfered with the fluorescence of DAPI staining. Here, we confirm in microspore cultures that enlargement is a marker for reprogramming. However, classical histology in this study gave clear evidence for cellular changes during reprogramming and for cell divisions occurring within microspores cultured at the mid or late uni-nucleate stage in genotype SM. This allowed us to follow the timing of nuclear divisions in the different pollen developmental pathways. Microspores at the late uni-nucleate stage, which are naturally scheduled to divide to form bi-cellular pollen grains by first pollen mitosis, could be induced by the pretreatment and the culture medium components to enter into the sporophytic pathway. At cell division, the nucleus moved to the centre and symmetric cell division occurred, demonstrating sporophytic development over asymmetric gametophytic development.

In TMS, we showed that responsive microspores in the tetrads were significantly different from their normal developmental pathway, with characteristic features such as vacuole formation and a light cytoplasm (as shown in Fig. 2) occurring at the time of enlargement. However, in some microspores containing dense cytoplasm, cell division occurred without any enlargement. Therefore, it seems that enlargement is a necessary component of reprogramming; however, some microspores enlarge further without being divided.

Apart from cassava, tomato is the only species in which androgenesis initiates both from the late uni-nucleate to early bi-nucleate stage and from the microspore mother cell to meiocyte stage. Seguí-Simarro and Nuez (2005) compared the two stages and suggested that the cytoskeleton may play a role in the developmental switch at both stages, since processes such as the establishment of cell polarity and cytokinesis are common to both stages and are mediated by the cytoskeleton. In the present study on cassava, it was found that the competent stages differed in the two genotypes, being the immature tetrad stage in one genotype (TMS) and the mid to late uni-nucleate stage in the other (SM). Further in-depth studies at the ultra-cellular level are being performed at present in order to find out the reason for these differences and possible common cellular features in the two genotypes with respect to reprogramming toward sporophytic development. Enlargement also occurred in the meiocytes of TMS. Four-nucleated syncytium formed prior to tetrad cellularization was clearly observed from the histological micrographs of tetrads in cold pretreated tetrads (Fig. 10D). Nuclear fusion may happen within these syncytial meiocytes and may develop into unreduced microspores as reported in cassava by Perera *et al.* (2013). Reprogramming in such unreduced microspores may give rise to MCSs and heterozygous diploid plants.

An important result of the present study was that the reprogramming occurred rapidly in both tetrads and microspores of TMS and SM. Microspore culture allowed us to detect the first changes towards sporophytic development within the first days of culture while in anther culture it took as long as 8 weeks to detect the first nuclear division in the microspores at the mid or late uni-nucleate stage (Perera *et al.* 2014). This clearly demonstrates the benefits of microspore culture. Multiple cell divisions occurred within 1 week in both genotypes forming MCSs. The current study clearly demonstrates that the thick microspore wall of cassava is no barrier for induction and further development of the MCS. When the microspore enlarges, and the sculptured particles of the exine move away from each other, creating spaces among the particles, this weakens the structure (as shown in Fig. 12E). Cell division in the MCS creates further pressure on the microspore wall, and then the cells are pushed out through the aperture, as shown by SEM (Fig. 7E and F), light microscopy (Fig. 11F) and transmission electron microscopy (TEM) (Fig. 12F). Given that the cassava microspore is multi-apertured, as shown in the SEM and histology micrographs, the pressure created by multiple cell divisions makes it easy to rupture the exine and release the MCS.

The NLN culture medium showed a negative response, indicating a specific induction effect of the NLNS medium (Kim *et al.* 2008). Brassica species efficiently give rise to microspore embryos in NLN or modified NLN media (Swanson 1990). The NLNS medium found to be superior in this study was additionally supplemented with potassium iodide (0.83 mg L^−1^), which, therefore, may be an essential nutrient for cassava. Cell divisions in cultured cassava microspores occurred only after supplementing the NLNS medium with extra amounts of NaFeEDTA and CuSO_4_·5H_2_O. A suitable form of ion supplement in the induction medium was found to be essential for further development of induced microspore embryos in tobacco (*Nicotiana tabacum*), barley and wheat (*Triticum* spp.) cultivars (genotypes), especially those providing few green plants via *in vitro* androgenesis (Novotný *et al.* 2000). Novotný *et al.* reported that, although Fe-EDTA was found to be a suitable form of ion in the induction medium, androgenesis was also induced on media containing non-chelated ion (Fe^2+^ and Fe^3+^ ions). Thus, the additional ion released from this complex may activate the cell division process in the induced or reprogrammed microspore in tetrads or the microspores. The presence of copper ions is reported to be effective in different ways in crops such as barley to increase green plant regeneration by decreasing starch production and increasing the quantity of internal membranes of plastids (Jacquard *et al.* 2009); however, the exact mode of action of these ions is still unclear.

## Conclusions

In conclusion, the present study clearly demonstrated the feasibility of reprogramming of microspores for sporophytic development in the tetrads of TMS 60444 and the mid or late uni-nucleate microspores in SM 1219-9. NLNS medium supplemented with NaFeEDTA and CuSO_4_·5H_2_O turned out to be beneficial for cell division in both genotypes. The light and fluorescent microscopic and SEM and TEM studies generated evidence for external and internal structural changes during the process of reprogramming. As in other species, microspore enlargement was found to be a valuable marker for reprogramming but with limits. Furthermore, the critical stage in microspore tetrads was characterized in detail and related to microspore reprogramming. The reason was attributed to the low frequency of reprogramming, and the presence of non-responsive microspores among the responsive ones was found to be related to exine synthesis and the viability of the microspores in tetrads. The present study clearly demonstrated that reprogramming is much faster in microspore culture than in anther culture of cassava, which paves the way for the development of an efficient technique for the production of doubled haploids in cassava. This is the first detailed report of microspore reprogramming at the tetrad stage and the first report on microspore embryogenesis induction in isolated microspores of cassava with analytical evidence.

## Sources of Funding

Our work was funded by the Bill & Melinda Gates Foundation, USA through grant ID no. OPPGD1483.

## Contributions by the Authors

P.I.P.P. was involved in designing all the experiments of this research, instructing the methodologies to be used under each experiment, data handling, manuscript preparation and submission. C.A.O. provided technical assistance for conducting the *in vitro* culture experiments and the histological analysis. B.D. managed the ordering of the chemicals. P.E.M.O. provided the technical support for fluorescent microscopic analysis.

## Conflicts of Interest Statement

None declared.
